# Neurotrophic Keratitis Following Vitrectomy Surgery: A Case Report

**DOI:** 10.7759/cureus.77372

**Published:** 2025-01-13

**Authors:** Jorge Saucedo, Fredy Shaid Lozano Feria, Andrés Ramírez

**Affiliations:** 1 Ophthalmology, Hospital Central Sur de Alta Especialidad, Mexico City, MEX

**Keywords:** diabetes type 2, herpetic keratitis, keratopathy, neutrotrophic keratitis, refractory corneal ulcer

## Abstract

Neurotrophic keratitis is a rare corneal degenerative disorder characterized by decreased sensory innervation, leading to epithelial defects and impaired wound healing. This report presents a case of a 60-year-old female with a history of type 2 diabetes and prior herpes infection who developed neurotrophic keratitis following ocular surgery for proliferative diabetic retinopathy. Initial treatment targeted infectious keratitis; however, persistent symptoms prompted a revised diagnosis. Management with autologous serum drops resulted in partial resolution of the epithelial defect but left a central leucoma and severely reduced visual acuity. This case highlights the need for early diagnosis and targeted therapy to prevent irreversible corneal damage in patients with high-risk profiles.

## Introduction

Neurotrophic keratitis is a rare degenerative disorder of the cornea caused by a decrease in sensory innervation. This impairment can arise anywhere along the pathway of the fifth cranial nerve, from its nuclei to the terminations of the nasociliary branch, resulting in varying degrees of corneal hypoesthesia [1].

Although the disease has an estimated prevalence of only 5 cases per 100,000 individuals, numerous risk factors can contribute to nerve damage. Herpetic keratitis is the most common underlying cause, with up to 6% of affected individuals subsequently developing neurotrophic keratitis. Similarly, corneal damage resulting from surgical procedures, burns, topical medications, and diabetes are significant contributing factors [2].

## Case presentation

We present the case of a 60-year-old female patient with a long-standing history of type 2 diabetes and a previous episode of facial herpes, without ocular involvement, over 20 years ago. Her medical history also included proliferative diabetic retinopathy complicated by tractional retinal detachment, for which she underwent phacoemulsification and vitrectomy surgery with silicone oil placement.

During the first postoperative week, the patient showed satisfactory recovery, achieving a visual acuity of 20/80 on the Snellen chart. However, on postoperative day 10, she presented to the clinic with complaints of decreased visual acuity and the sudden onset of ocular pain 36 hours prior, rated as 5/10 on the Numeric Visual Scale (NVS).

On examination, her visual acuity had deteriorated (finger count). Slit lamp examination revealed a fluorescein-stained, central epithelial lesion with irregular borders, measuring approximately 2.5 × 3.0 mm. The patient was diagnosed with infectious keratitis and prescribed a preservative-free ocular lubricant, topical moxifloxacin 0.5%, as well as a cycloplegic.

**Figure 1:**
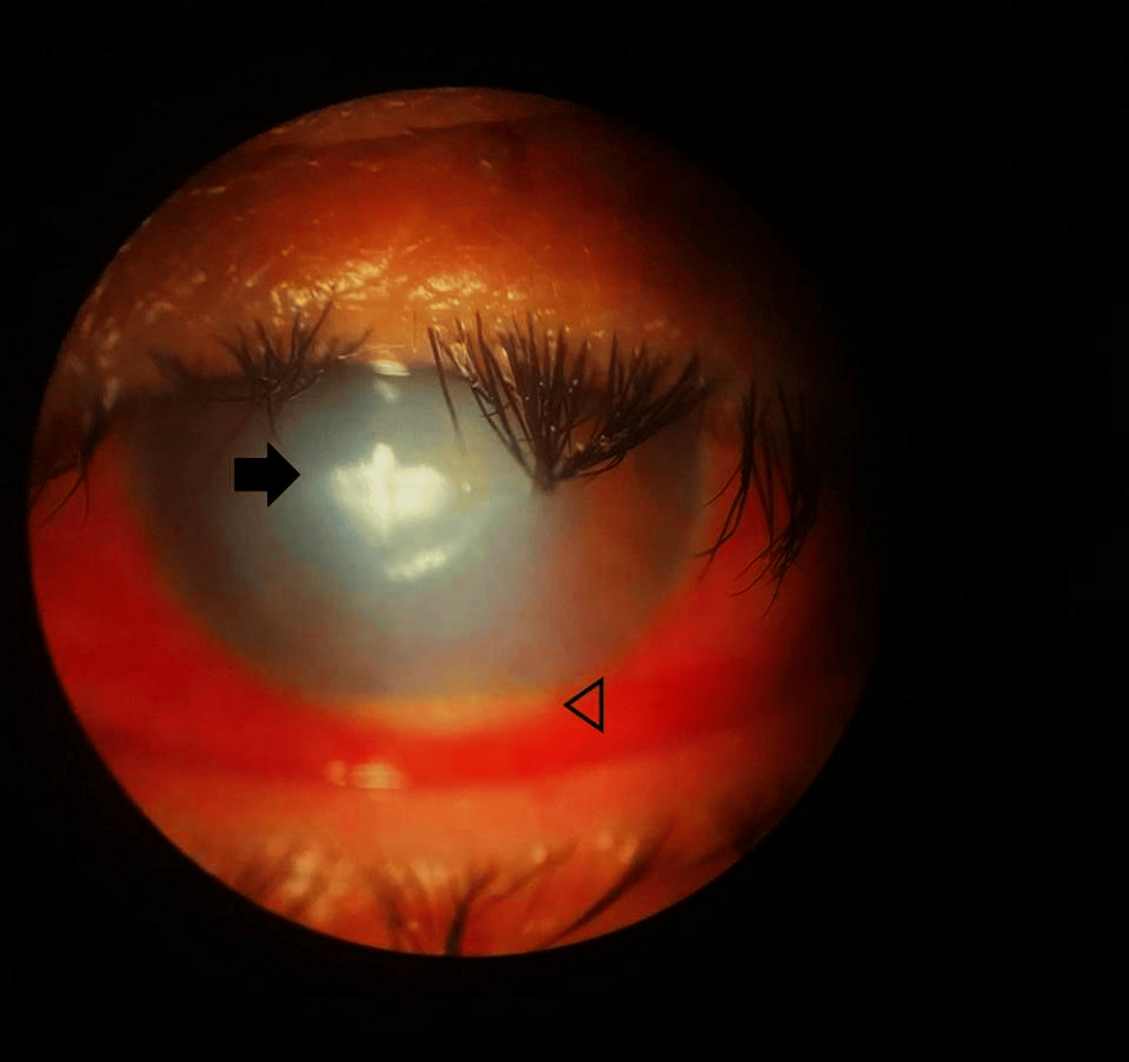
Clinical picture showing a central epithelial and stromal opacity (arrow). In the anterior chamber, a 0.5 mm hypopyon is shown (arrowhead). The central corneal defect and the anterior chamber inflammation make visibility inside the eye difficult

**Figure 2:**
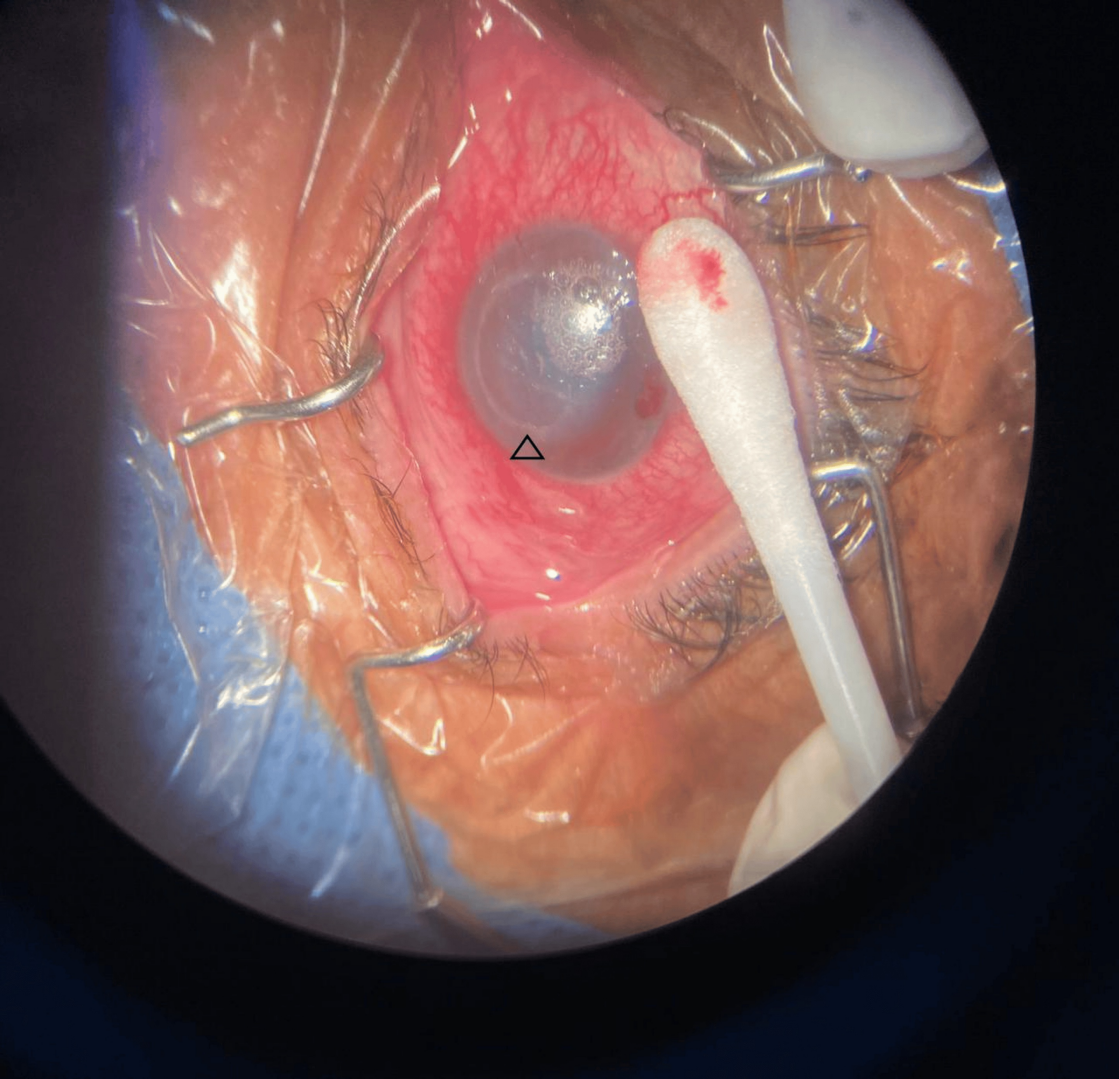
Clinical picture during corneal scraping and aqueous humor sampling. A central epithelial defect with well-defined margins (arrowhead)

One week later, the patient reported increased ocular pain, now rated 8/10, accompanied by further reduction in visual acuity to light perception. The epithelial lesion had enlarged to approximately 3.5 × 3.5 mm, and slit-lamp examination revealed hypopyon and anterior chamber cellularity (Figure 1). Corneal scraping and aqueous humor sampling were performed for Gram staining, culture, and immunoassays to rule out herpes simplex and varicella-zoster (Figure 2). Treatment was escalated to fortified amikacin (40 mg/mL), fortified vancomycin (5%), and topical insulin (1 IU/mL every six hours).

**Figure 3:**
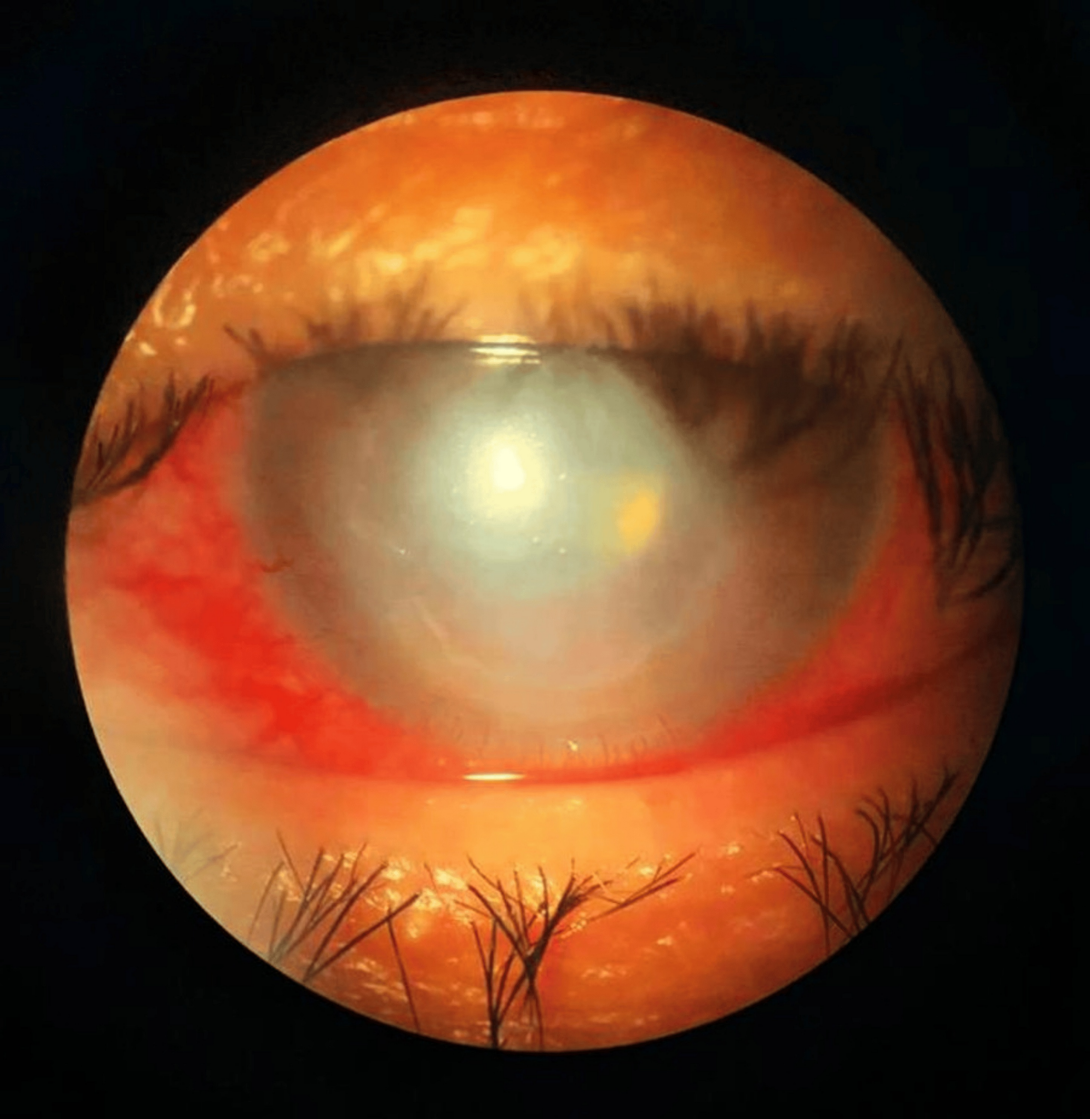
Clinical picture in which the central epithelial defect is still well defined. However, the hypopyon inside the anterior chamber is no longer present, and the visibility inside the anterior chamber has drastically improved

Despite one week of treatment, microbiological studies returned negative, but ocular pain persisted at 8/10. Neurotrophic keratitis was suspected, leading to the discontinuation of reinforced antibiotics. Treatment was switched to 50% autologous serum drops. One week later, the patient reported significant pain relief, with diminished anterior chamber inflammation and improvement of the epithelial defect (Figure 3). By the second week of treatment, the epithelial defect had resolved.

In spite of prompt intervention, the patient developed a 3.0 × 3.0 mm central leucoma. The final visual acuity was limited to light perception.

## Discussion

The pathophysiology of neurotrophic keratitis involves a loss of sensitivity in the corneal surface due to damage to the trigeminal nerve, which supplies the subepithelial corneal plexus. This plexus is located between Bowman’s membrane and the basal cells of the corneal epithelium. In conditions involving nerve damage, such as herpes simplex virus (HSV) infections, the loss of subepithelial plexus density leads to hypoesthesia, disruption of trophic factors that mediate epithelial cell migration, and impaired nervous communication essential for maintaining the tear film's quality and quantity [3].

Patients with neurotrophic keratitis are often asymptomatic or present with nonspecific symptoms such as foreign body sensation, blurred vision, or red eyes. However, biomicroscopy can reveal corneal alterations, including epithelial cell loss and reduced regenerative capacity. Mackie’s classification divides the disease into three stages based on slit-lamp findings: Stage 1: Superficial punctate keratitis, stromal scars, or superficial neovascularization; Stage 2: Persistent epithelial lesions or stromal edema; Stage 3: Corneal ulceration progressing to perforation or lysis [4].

In contrast, the Neurotrophic Keratopathy Study Group classification incorporates parameters that precede slit-lamp findings and includes the following phases: Phase 1: Sensory alterations without keratopathy; Phase 2: Epitheliopathy or punctate keratopathy without corneal haze; Phase 3: Persistent or recurrent epithelial defects without haze; Phase 4: Recurrent epithelial defects with corneal haze; Phase 5: Recurrent epithelial defects with corneal ulceration; Phase 6: Corneal perforation [4].

Assessment of neurotrophic keratitis requires the documentation of epithelial lesions via slit-lamp examination, corneal sensitivity testing with tools such as an air esthesiometer or Cochet-Bonnet esthesiometer, evaluation of tear film function, and microbiological studies to rule out infections [5].

Currently, no specific treatment exists for neurotrophic keratitis. Management in stage 1 focuses on protecting the corneal surface and improving lubrication using preservative-free artificial tears and therapeutic soft contact lenses. In stages 2 and 3, treatment aims to promote re-epithelialization and prevent further damage [5].

In cases of stromal melting, especially in the context of neurotrophic keratitis, treatment strategies aim to manage inflammation, prevent infection, and promote healing. Topical insulin and topical collagenase inhibitors (such as N-acetylcysteine) are used to inhibit collagenase activity, helping prevent stromal thinning and melting, which are common complications in advanced stages of neurotrophic keratitis. Systemic agents such as tetracycline or medroxyprogesterone exhibit anti-inflammatory properties, modulating the immune response and reducing the risk of corneal damage [6].

Topical antibiotics are essential in stages 2 and 3 to reduce the risk of infection, which could exacerbate the condition and lead to further complications [7]. Topical nerve growth factor (NGF), derived from Adeno-Associated Virus rh10 (AAV.rh10), is a promising treatment option shown to promote nerve regeneration in the subepithelial plexus in animal models, thereby enhancing corneal healing [8].

The use of autologous serum has been extensively studied for recurrent epithelial defects and early stages of neurotrophic keratitis (stages 1 and 2). For more severe cases, 50% autologous serum or plasma rich in growth factors may offer greater benefits [9,10]. Recent studies have highlighted the synergistic role of substance P and insulin-like growth factor 1 (IGF-1) in mediating corneal cell migration and re-epithelialization, suggesting the potential therapeutic benefit of exogenous substance P in this pathology [11].

These treatments are typically combined to address the multifaceted aspects of neurotrophic keratitis, aiming to promote healing, reduce inflammation, and prevent infection, thereby preserving corneal integrity and vision [12].

For advanced cases (Mackie stages 2 and 3), surgical interventions may be necessary. Options include tarsorrhaphy, amniotic membrane transplantation, conjunctival flaps, or corneal neurotization, the latter involving supraorbital or supratrochlear nerve transplantation. Corneal neurotization has shown promising results in recent years [12].

This case underscores the importance of prompt intervention for corneal ulcers to prevent severe complications, such as visually compromising leucomas or corneal perforation. It also highlights the need to suspect neurotrophic keratitis in patients with diabetes and a history of herpes infection.

## Conclusions

Neurotrophic keratitis remains a challenging condition due to its insidious onset, nonspecific symptoms, and potential for severe visual impairment if left untreated. This case highlights the critical importance of early recognition, especially in patients with known risk factors such as diabetes and a history of herpes infection. Prompt diagnosis and tailored management, including the use of advanced therapies like autologous serum, can mitigate progression and improve outcomes. Awareness and a multidisciplinary approach are essential to preserve vision and prevent complications such as corneal perforation or leucoma.
